# Genomic approaches for improving grain zinc and iron content in wheat

**DOI:** 10.3389/fgene.2022.1045955

**Published:** 2022-11-08

**Authors:** Chandan Roy, Sudhir Kumar, Rakesh Deo Ranjan, Sita Ram Kumhar, Velu Govindan

**Affiliations:** ^1^ Department of Genetics and Plant Breeding, Agriculture University, Jodhpur, Rajasthan, India; ^2^ Department of Plant Breeding and Genetics, Bihar Agricultural University, Bhagalpur, Bihar, India; ^3^ International Maize and Wheat Improvement Center (CIMMYT), Mexico City, Mexico

**Keywords:** malnutrition, QTL mapping, GWAS-genome-wide association study, speed breeding, new breeding techniques (NBTs), biofortification, genomic selection

## Abstract

More than three billion people worldwide suffer from iron deficiency associated anemia and an equal number people suffer from zinc deficiency. These conditions are more prevalent in Sub-Saharan Africa and South Asia. In developing countries, children under the age of five with stunted growth and pregnant or lactating women were found to be at high risk of zinc and iron deficiencies. Biofortification, defined as breeding to develop varieties of staple food crops whose grain contains higher levels of micronutrients such as iron and zinc, are one of the most promising, cost-effective and sustainable ways to improve the health in resource-poor households, particularly in rural areas where families consume some part of what they grow. Biofortification through conventional breeding in wheat, particularly for grain zinc and iron, have made significant contributions, transferring important genes and quantitative trait loci (QTLs) from wild and related species into cultivated wheat. Nonetheless, the quantitative, genetically complex nature of iron and zinc levels in wheat grain limits progress through conventional breeding, making it difficult to attain genetic gain both for yield and grain mineral concentrations. Wheat biofortification can be achieved by enhancing mineral uptake, source-to-sink translocation of minerals and their deposition into grains, and the bioavailability of the minerals. A number of QTLs with major and minor effects for those traits have been detected in wheat; introducing the most effective into breeding lines will increase grain zinc and iron concentrations. New approaches to achieve this include marker assisted selection and genomic selection. Faster breeding approaches need to be combined to simultaneously increase grain mineral content and yield in wheat breeding lines.

## Introduction

Minerals are important components for physical and mental health development in humans. Malnutrition from micronutrient deficiencies, also known as “hidden hunger,” is one of the most challenging health issues globally and particularly in developing countries. Dietary deficiencies of zinc (Zn), iron (Fe), iodine, and vitamin A are most prevalent among children and women. Worldwide, more than three billion people are affected by zinc (Zn), iron (Fe), and vitamin-A deficiencies (Raemaekers, 1988; Beal et al., 2017), with such nutrient deficiencies being particularly high among people in Asia and Sub-Saharan Africa. Fe is an essential part of hemoglobin and myoglobin and being directly involved in oxygen transport, enzymatic functions, energy production and DNA synthesis. Fe deficiency causes anemia (https://www.who.int/health-topics/anaemia); children below 5 years of age (40%) and women at reproductive or lactating stages (30%) are more anemic. Likewise, Zn is a ubiquitous element for every living organism including human beings, acting as a co-factor for more than 300 enzymes and proteins at the cellular and sub-cellular levels during nucleic acid production, metabolism, cell division and differentiation, and the immune system. Zn deficiency impairs physical growth and development and the proper functioning of the immune and reproductive systems and mental acuity, as well as increasing child mortality. On average 20% of women and children are Zn deficient, with a high prevalence in low- and middle-income groups and even among adult men (Gupta et al., 2020b). Another study reported 31.3% Zn deficiency among the children of higher income groups in western Europe and no significant difference was observed between children of the different socio-economic groups (Vreugdenhil et al., 2021). Food supplements and fortification and more diverse diets can help to address micronutrient malnutrition. Iron and zinc supplements, for example, have substantially reduced diarrhea and anaemia in affected segments of the population (Gupta et al., 2020b). Food fortification for vitamin-A and iodine deficiency through pharmaceutical products and iodized salt have reduced related deficiencies, but such products may be unaffordable for low-income families, particularly in developing countries. Reduced consumption of vegetables and fruits is observed due to lower purchasing power in many farm households in Asia and Sub-Saharan Africa; now the COVID-19 outbreak substantially reduced the income of poor households that cut down on their intake of fruits, vegetables, and animal-based foods and increased their dependence on staple grains (Heck et al., 2020). In 2022, restrictions in the movement of farm produce, disrupted supply systems, and continued economic fall-out due to the COVID-19 pandemic and Russia-Ukraine conflict jeopardized food supply chains, including distribution to Central Asia and Africa, and triggered food prices, augmenting global hunger and malnutrition (https://www.worldbank.org/en/topic/agriculture/brief/food-security-update). Increasing nutrient density and bioavailability in staple food crops, as well as their production and distribution at affordable prices, can greatly improve the nutrition of low-income groups.

“Biofortification” refers to increasing essential mineral and vitamin content and bioavailability in edible parts of staple food crops, either through conventional breeding and/or biotechnological interventions, as well as through fertilizer. “Golden rice” is a successful example of improving the beta-carotene content in rice through the transformation of three bio-synthetic pathway genes: phytoene synthase (*psy*), phytoene desaturase (*crtI*) and lycopene β-cyclase (*lcy*) (Ye et al., 2000). Biofortification in staple food crops has progressed through initiatives such as Harvest Plus, the Grand Challenge in Global Health, the India Biofortification Programme, Scaling Up Nutrition (SUN), and Global Alliance for Improved Nutrition (GAIN), among others, and has gained global and local recognition. Numerous countries (Bangladesh, Brazil, China, Colombia, India, Indonesia, Malawi, Nigeria, Pakistan, Panama, Rwanda, Uganda, and Zambia) have included biofortification in their national health and development policies (Virk et al., 2021). Efforts also have been made to speed the seed production and distribution of biofortified crop varieties to reach resource-poor farmers in remote areas. The government of India has proposed the inclusion of fortified wheat, rice, edible oil in mid-day meal programs, the public distribution system and integrated child development program (https://www.livemint.com/Politics/91RAsPJFJLykDywTPHRCeI/Fortified-MidDay-meals-by-December-2019-to-fight-malnutriti.html).

Wheat provides 25% of calories in human diets worldwide and 60% in Central and West Asian countries (Cakmak, 2008). The wheat endosperm is rich in starch but poor in minerals, particularly Zn, Fe, and vitamins (Cakmak and Kutman, 2018). Moreover, minerals and bioactive components of the wheat grain are concentrated in the aleurone layer, which is removed during milling, so the remaining flour generally contains only small amounts of minerals and vitamins. The average Zn concentration in the white flour is around 8 mg per kg, falling short of the daily recommended dietary allowance (RDA) of from 9–19 mg per day for adults (Cakmak, 2007; Gillies et al., 2012; Cakmak and Kutman, 2018). The low mineral contents in rice and wheat is due to the deficiencies of these substances, particularly of Zn, in the soils of most of the areas where the crops are grown. Finally, breeding for high yielding wheat genotypes since the mid-20th century has reduced genetic variation for grain Zn and Fe in modern cultivars from the levels found in landraces and genotypes released during Green Revolution (Debnath et al., 2021).

Through agronomic and genetic interventions, mineral density can be enhanced (Cakmak, 2008). Genetic biofortification is the most sustainable and cost-effective approach to alleviate micronutrient malnutrition among lower-middle income consumers. Based on estimated average micronutrient requirements; average staple food crop intakes; mineral losses incurred during harvesting, processing, and storage; and micronutrient bioavailablility, a minimum target levels have been set for different minerals in staple food crops (Bouis et al., 2011). To meet out 30 and 40% of estimated average requirements for Fe and Zn, respectively for children and adult women, the target levels of 59 ppm for Fe and 38 ppm for Zn are set in wheat grain, considering the baseline of 30 ppm for Fe and 24 ppm for Zn. Large-scale screening of germplasm, identification of genotypes, mapping of underpinning genomic regions, and their use to develop high-yielding, disease resistant, biofortified wheat genotypes are the major focuses of genetic biofortification research. Wild relatives carry higher levels of Zn and Fe in their grain than cultivated wheat genotypes. The locus *Gpc-B1* for grain protein, Zn, and Fe was detected and fine mapped in *Triticum durum* ssp *dicoccoides* and can serve as a source to enhance these traits in cultivated wheat (Uauy et al., 2006). Genome sequence information of *T. aestivum* cv. Chinese Spring (Mayer et al., 2014) and a highly annotated chromosome level genome sequence (Ref Seqv1.0; Appels et al., 2018) can be used for gene discovery, cloning, and functional analysis of genome targeting to grain Zn and Fe. In this article we review the status of wheat biofortification and how genomic resources can be used in breeding for biofortified wheat genotypes.

## Agronomic vs*.* genetic biofortification

Initial research on agronomic biofortification to increase grain Zn in wheat took place in Turkey (Cakmak et al., 1996; Cakmak et al., 1999). Mineral density can be modulated by soil Zn and Fe status and their availability for the crop. Soil zinc and iron availability depends on edaphic factors such as soil physical and chemical properties, micronutrient availability, pH, the status of soil moisture and organic matter, grain filling duration, and the timing of senescence (Cakmak and Kutman, 2018). Irrespective of this, wheat grain zinc and iron concentrations can be increased by applying these elements directly to wheat (Yilmaz et al., 1997). Zn and Fe can be applied in inorganic or organic forms but the most commonly used are ZnSO_4_ and FeSO_4_, due to their ready availability and low cost. Soil and foliar applications of zinc fertilizer increase grain Zn density in wheat and can positively affect agronomic parameters such as the number of tillers, thousand kernel weight, chlorophyll content, and biological yield (Rizwan et al., 2018; Dimkpa et al., 2020). Hussain et al. (2012b) reported that soil applications of Zn increased whole grain zinc concentrations up to 95% and grain yield by 29%. However, foliar application was found to be more effective than soil application to increase zinc and iron concentrations in wheat. Foliar application is also advantageous under stress conditions, particularly drought, to avoid impairment of mineral uptake due to lack of soil moisture. Foliar applications of ZnSO_4_ @ 0.5% and FeSO_4_ @ 1% were more effective than applying to soil alone or both soil and foliar applications (Ramzan et al., 2020), and foliar application during grain filling resulted in a more effective translocation of zinc to the grain. Spraying zinc fertilizer at the grain development stage improved grain zinc concentration by 68% (Zhang et al., 2010). Unlike for zinc, iron fertilization of soils is not effective at increasing grain iron concentrations (Gupta, 1991), but in several studies the foliar application of iron increased grain Fe concentration up to 28% (Zhang et al., 2010); 21% (Pahlavan-Rad and Pessarakli, 2009), and 14% (Aciksoz et al., 2011b). Application of N-fertilizers along with FeSO_4_ increased iron concentrations in shoots as well as in the grain (Aciksoz et al., 2011b; Singh et al., 2018). Previous reports showed that N application increased the secretion of phytosederophore that chelate Fe in the soil and Yellow Stripe 1 protein act as Fe transporter (Aciksoz et al., 2011a). Seed priming is another way to deliver minerals to plants. Zinc also improves coleoptile and radical growth and germination in wheat (Ozturk et al., 2006). The recent practice of applying nano particles (NP) to the soil is becoming popular as a cost effective, environmentally-friendly approach to reduce fertilizer losses and raise productivity and profitability. Applying ZnO-NP and Fe-NP in wheat enhanced grain yield, biomass, chlorophyll content, and drought tolerance (Dimkpa et al., 2020). Nano zinc and iron treatments significantly increased Zn and Fe concentration in the roots, shoots, and grain (Rizwan et al., 2018; Dimkpa et al., 2020). Applying ZnO-NP or Fe-NP alone or coated with NPK can reduce farmers’ costs and, in case of zinc, it is cheaper than ZnSO_4_.

Whereas, foliar and soil applications of Zn and Fe must be repeated in each crop cycle, genetic biofortification provides a long-term solution against micronutrient malnutrition. Genetic biofortification is complex, involving several physiological pathways and proteins right from soil uptake of minerals to their accumulation in the grain. Targeting the genes or proteins that play key roles in mineral sequestration may increase grain Zn and Fe. The two major approaches that may be used are selective plant breeding and transgenic breeding, to develop biofortified crop genotypes. Transgenic approaches and outputs are subject to stringent policies in many countries and their use is restricted or prohibited, so this paper focuses on selective breeding. Mainstreaming biofortification as a selection priority in breeding programs—along with yield, disease resistance, end-use preferences, and environmental adaption—is paramount, and includes the use of micronutrient dense parental lines and setting grain micronutrient target levels. The review consists of three major sections: the first section deals with physiological and molecular mechanism of uptake, translocation and grain sequestration of Zn and Fe; second section deals with breeding for grain zinc and iron including mapping of gene(s) and QTLs; and the third section deals with new breeding techniques which need to be adopted with conventional breeding.

## Uptake of soil zinc and iron through roots

Complex physiological and metabolic processes are associated with the uptake and accumulation of Zn and Fe into the grain. Broadly, there are three kinds of mineral uptake mechanisms: 1) direct uptake of Zn^2+^ and Fe^2+^ molecules *via* the root system with the help of ZRT-IRT like proteins; 2) where Zn^2+^ and Fe^3+^ molecules are chelated first and then taken up through roots; and 3) a combination of 1 and 2. Cereals and millets (rice, maize, wheat, barley, pearl millet, among others) use mechanism 2 to extract Fe and Zn from the rhizosphere into the root system, where secretion of phytosiderophores (PS), proteins of the Mugineic acid (MA) family, help in chelating Fe and Zn and form a complex of Fe^3+^-MA or Zn^2+^-MA (Marschner and Romheld, 1994; Nozoye et al., 2015). The role of MA is well understood in Fe uptake and transport in rice and barley, but also showed its affinity towards molecules like Zn and Cu. In zinc-deficient wheat plants, more MA was released (Cakmak et al., 1994). Methionine acts as a precursor of MA synthesis, which is converted to S-adenosyl-L-methionine (SAM) with the help of the enzyme SAM synthetase (Mori and Nishizawa, 1987). In a subsequent reaction, SAM is converted to nicotianamine (NA) by NA synthase (NAS) followed by 3″-keto acid and 2′-deoxymugineic acid (DMA) by NA aminotransferase (NAAT) and DMA synthase (DMAS), respectively (Shojima et al., 1990). In some species, DMA is further converted to MA. In graminaceous crops MA is commonly produced, whereas NA is most common in non-graminaceous crops. In wheat, MA producing genes such as *TaNAS*, *TaNAAT*, and *TaDMAS* were identified (Pearce et al., 2014). MA secreted in the root zone chelates iron along with zinc, manganese (Mn), and copper (Cu). The complexes of Fe^3+^-MA or Zn^2+^-MA are taken up by roots. This mechanism is well established for Fe uptake in rice, barley and maize, but little is known about the genetic mechanism of metal uptake and its transport in wheat. Using the knowledge of model crop species, it is possible to identify the candidate genes in wheat of potential use for breeding.

The gene family “transfer of MAs” (*TOM*) plays important role in the uptake of soil iron and its translocation. *TOM1* was first identified in rice and barley as facilitating MA secretions from roots to soil (Nozoye et al., 2011) and their transport. *TOM2* and *TOM3* are homologous to *TOM1* and transport PS into the plant. Over expression of *TOM2* using a *GUS-*promoter in rice allowed scientists to locate it in roots, shoots and seeds, with the highest expression in basal plant tissues (Nozoye et al., 2015). Several zinc-induced facilitator (*ZIFL*) genes have been identified and their role in zinc homeostasis assessed. *ZIFL* acts as an efflux transporter of vacuolar NA. In rice, *ZIFL* transports MA synthesized in the roots. In wheat, 15 *Ta*ZIFL proteins distributed on chromosomes 3, 4, and 5 have been characterized; genes like *TaZIFL2.3*, *TaZIFL4.1*, *TaZIFL4.2*, *TaZIFL5*, *TaZIFL6.1*, and *TaZIFL6.2*, were upregulated in roots under zinc and Fe deficient conditions (Sharma et al., 2019).

Other than MA, nicotianamine is produced by roots that also help in chelating soil Fe and Zn. *NAS* increases the production of NA and DMA, which improves the uptake and translocation of chelated metals. Overexpression of rice nicotianamine synthase genes, the *OsNAS2* gene in wheat, increases the uptake and translocation of grain zinc in shoots as well as the grain (Singh et al., 2017).

## Transport of zinc and iron

A complex mechanism of metal transport, chelation and sequestration helps plants to avoid metal toxicity. Plants produce several transporter proteins varying in their substrate, expression, and locations. Colangelo and Guerinot (2006) provide a detailed discussion of the P_1B_-ATPase family, the CDF family as metal efflux proteins, ZIP, yellow stripe-1 (YSL), the natural resistance associated macrophage protein (NRAMP), and the copper uptake proteins (COPT) as metal uptake proteins. Heavy metal transporting P type ATPase (HMA), a protein of the P_1B_-ATPase family, act as a Zn, Cd, and Pb transporter. *Arabidopsis* mutants of *hma2* and *hma4* showed lower Zn uptake than the wild type (Hussain et al., 2004). HMA helps Zn to move from roots into shoots and plays a role in xylem loading and unloading. In wheat, 32 HMA protein-producing genes were detected on chromosomes 2A, 2B, 2D, 4A, 4D, 5A, 5B, 5D, 6A, 6B, 6D, 7A, 7B, and 7D (Zhou et al., 2019). Overexpression of the *TaHMA2* gene in wheat increased the Zn concentration in the shoot as well as in the grain; however, grain Zn concentration was limited to the embryo and aleurone layer (Tan et al., 2013). Similarly, metal tolerance protein (MTP) is another transmembrane metal transporter protein of the CDF family located in the vacuole membrane of roots, shoots, and leaves and helps in Zn transport into the vacuole. Knocking out the *mtp1* gene in *Arabidopsis* reduced Zn accumulation in various plant tissues (Desbrosses-Fonrouge et al., 2005). NRAMP is another protein located in the vacuole membrane and which takes active part in Fe transport. Eight homologues of *TaNRAMP* genes have been identified in wheat (Borrill et al., 2014). Other genes known as vacuolar iron transporter (*VITs*) genes help transport of iron into the grain and offer a potential target for iron biofortification. Wheat has two functional *VIT* genes, *TaVIT1* and *TaVIT2* on chromosomes 2 and 5, each with three homeologs from the A, B, and D genome. Overexpression of *TaVIT2* increased grain iron 2-fold (Connorton et al., 2017).

Several proteins belong to the yellow stripe (YSL) family, a complex of transporter proteins that help in the uptake of chelated metals in the soil into roots and their subsequent transport to stems, leaves, and grain. First detected in maize, *YSL-1* is involved in the uptake of metals that form complexes with PS or NA and its role in Fe^3+^-MA transport has been well established (Curie et al., 2001). Rice genes *OsYSL15* express in the seed and are involved in seed germination, while *OsYSL2* plays an important role in phloem transport and transport into the seed (Nozoye et al., 2015). Likewise, zinc-induced transporter family (*ZIP*) proteins are involved in metal uptake and transport. In wheat, 14 and 19 members of *YSL* and *ZIP* gene families have been characterized in up and down regulation during senescence and which play key roles in metal transport from the cytoplasm to the phloem and the phloem to the grain (Pearce et al., 2014). Several *YSL, ZIP*, and *NRAMP* homologs are involved in the uptake, transport, and remobilization of Fe and Zn in wheat (Table 1). Overexpression of the specific transporter protein using a tissue-specific promoter could be a target area for research to increase the grain mineral concentrations.

**TABLE 1:** Summary of genes involved in uptake, transport and grain accumulation of Zn and Fe in wheat.

Activity	Genes	Plant organs	References
Minerals uptake	*NAAT2-D, DMAS1-B, TOM, ZIP1, ZIP3, ZIP6, ZIP7, ZIP9, ZIP13, TaVTL1, TaVTL2, TaVTL5, TaZIFL2.3, TaZIFL4.1, TaZIFL4.2, TaZIFL5, TaZIFL6.1* and *TaZIFL6.2, TaYS1A, TaYS1B, TaYSL3, TaYSL5, *and* TaYSL6, TaNRAMP5*	Roots	Ajeesh-Krishna et al. (2020); Gupta et al. (2020a); Kumar et al. (2018); Sharma et al. (2019); Sharma et al. (2020)
Mineral transport	*ZIP1, ZIP3, ZIP6, ZIP7, ZIP9, ZIP10, ZIP13, ZIP15, TaHMA2, TaNRAMP3, TaNRAMP5, TaCNR2 (Cell number regulator 2), TaYSL1A, TaYSL1B, TaYSL5, TaYSL12,* and *TaYSL19, TaVIT1* and *TaVIT2*	Stem, leaves	Ajeesh-Krishna et al. (2020); Connorton et al. (2017); Kumar et al. (2018); Peng et al. (2018); Qiao et al. (2019); Tan et al. (2013)
Mineral accumulation into grain	*TaFer1, TaFer2, TaMTP1-8A, TaNRAMP3, ZIP1, ZIP3, ZIP7, ZIP10, ZIP15*	Spikletes, grains, aleurone layer	Ajeesh Krishna et al. (2020); Borg et al. (2012); Vatansever et al. (2017)

Italic values indicates the gene name

## Increase Zn and Fe in wheat endosperm

Minerals deposition in grain occurs through the direct uptake of Zn/Fe by roots at grain filling stage, or remobilization of stored Zn/Fe from leaves and stems. Studies in rice to understand the contribution through continued root uptake and remobilization of stored minerals into the grain observed that minerals are accumulated *via* both processes. Under conditions of adequate minerals in the soil, continued root uptake plays a major role in Zn/Fe deposition into the grain while, under mineral deficient conditions, remobilization of stored Zn/Fe from leaves and stems contributes more to rice grain mineral content (Sperotto, 2013). A similar result was observed for grain Zn accumulation in winter wheat by Liu et al. (2019a), also reported a critical value for soil Zn availability (7.15 mg kg^−1^ DTPA Zn concentration) for wheat, above which the direct uptake of soil Zn during grain filling is predominant and below which remobilization is the major source of Zn in the grain. Remobilization of Zn/Fe depends on the xylem to phloem loading and mobility. In rice, grain Zn concentrations depend on plants’ ability to redistribute Zn from older leaves and stems, as well as phloem mobilization of Zn (Wu et al., 2010). Significant contributions of the flag and penultimate leaves to wheat grain yield have been observed (Roy et al., 2021a), but there are no detailed studies concerning the proportionate remobilization of Zn and Fe from different wheat plant parts. Uauy et al. (2006) observed an abundance of Zn, Fe and proteins in wheat flag leaves during grain filling, in plants carrying the *Gpc-B1* locus.

Micro elemental analysis revealed higher concentrations of Zn, Fe and other bioactive elements in the embryo and grain aleurone layer; achieving localized increases of mineral concentrations in the endosperm is challenging. To better understand mineral translocation and deposition in the grain, experiments in model crop plants identified key genes. Ferritin is a Fe storage protein located in the plastid and readily bio-available. Enhancing *FERRITIN* gene expression is important for Fe-biofortification. Genetically transforming rice using the soybean *FERRITIN* gene under the seed-specific promoter gene *Glu-B1* (Goto et al., 1999) and the pea *FERRITIN* gene under the *Gt-1* promoter (Lucca et al., 1999) increased endosperm iron levels. The soybean *FERRITIN* gene under the control of the maize ubiquitin promoter was expressed in wheat, resulting in higher iron concentrations in both plant tissue and the grain, but with much higher levels in the former (Drakakaki et al., 2000). Wheat carries two ferritin genes, *TaFer1* and *TaFer2,* located in chromosomes 4 and 5 and each with three homoalleles in hexaploid wheat. Overexpression of the endogenous *TaFer1-A* gene targeting the endosperm raised wheat grain Fe levels by 50%–58% (Borg et al., 2012).

The endogenous vacuolar transporter (*TaVIT2*) gene using a promoter of the *GLU-1D-1* gene achieved a more than 2-fold increase in Fe concentrations in white flour (Connorton et al., 2017). In another attempt, the constitutive expression in bread wheat of the rice *OsNAS* gene that produces chelators such as nicotianamine (NA) and 2′-deoxymugineic acid (DMA) improved grain Fe and Zn concentrations (Beasley et al., 2019).

Effective genetic biofortification approaches for wheat should focus on increasing mineral uptake from the soil and translocation and remobilization into grains, combining genes for higher metal uptake and translocation (*NAS, NAAT, DMAS, TOM, YSL, ZIP etc.*) and genes that affect targeted transport to the endosperm (*VIT, FERITTIN etc.*). Singh et al. (2017) developed wheat transgenic lines using the rice *OsNAS2* and bean *PvFERRITIN* genes separately and combining both genes and observed increased grain Zn and Fe concentrations in all three cases. The *OsNAS2* gene was most effective; a grain of related transgenic wheat lines contained 93.1 ppm of Fe and 140.6 ppm of Zn (Singh et al., 2017) and there was a more than 2-fold increase in grain Fe, compared to a 1.6-fold increase with the combined expression of both genes. This differs from the case for rice, where the combined expression of *OsNAS2* and *FERRITIN* gave a 6-fold increase in grain Fe content (Trijatmiko et al., 2016), suggesting that the two genes may not be synergistic in wheat and the overexpression of endogenous genes in wheat could be an alternative for increasing endospermic mineral expression. However, the very low efficiency of transformation in wheat, compared with crops like rice and barley, and of transgene expression in hexaploid wheat needs to be considered.

## Increasing bioavailability of minerals

Minerals in cereal and legume grains are less bioavailable. The presence of phytic acid (PA), a phosphorus (P) storage protein in seed representing 65%–85% of seed P, acts as a chelator for cations of Ca^2+^, Mg^2+^, Zn^2+^, Mn^2+^, and Fe^3+^ and reduces their absorption in the intestine (Bhati et al., 2016). Thus, the bioavailability of grain Zn and Fe depends on proportionate phytic acid content in the grain or diet. The molar ratio of phytate: Zn/Fe can be used as a determinant of mineral bioavailability; an increase in the molar ratio indicates lower adsorption of Zn and Fe. Morris and Ellis (1989) determined critical ratios of phytate: Fe > 1 (Hallberg et al., 1989) and phytate: Zn > 15. Genotypes with lower phytic acid lines of PA: Zn < 0.4 and PA: Fe < 5 or 5–10 are preferable for biofortification (Gupta et al., 2022). Assessment of phytic acid in wheat genotypes showed higher genetic variability. Average PA in wheat grain ranges from 7.1 to 11.1 mg g^−1^, giving a molar ratio for PA: Zn from 24 to 41, in a set of 65 wheat genotypes from Pakistan (Hussain et al., 2012a). Another study involving 42 durum wheat genotypes showed PA variation from 0.462 to 0.952% and ranges for molar ratios of PA: Zn and PA: Fe of 16.9–23.6 and 12.1–29.6, respectively (Magallanes-Lopez et al., 2017). Wen et al. (2022) evaluated 330 wheat genotypes from CIMMYT, Mexico and reported 0.9%–1.72% range in PA content. Genome wide association studies revealed six stable genomic regions and the effect of four of the six region could reduce PA content from 1.21% to 1.13% which could potentially increase grain Zn and Fe bioavailability by 7%. In comparison, the PA content in *T. monococcum*, *T. turgidum* and *T. aestivum* was less in diploids than in tetraploids or hexaploids (Bilgrami et al., 2018). Seasonal variation of PA was also observed a significant difference in PA between tetraploids and hexaploids was recorded in the fall but was non-significant in the spring. Likewise, the PA: Zn molar ratio was less in *T. monococcum* (2.15), than *T. turgidum* (2.66) and *T. aestivum* (3.15) (Bilgrami et al., 2018). Understanding the physiological and metabolic pathways their underlying genes related to PA biosynthesis allow researchers to mitigate/reduce/control PA activity in grains. Phytic acid is produced during grain development and abscisic acid (ABA) and gibberellic acid (GA) regulates its accumulation during seed maturation. PA is synthesized from glucose-6-phosphate through a series of phosphorylation reactions by several inositol phosphate kinases (IPK) proteins. Genes such as *TaIMP, TaITPK1-4, TaPLC1, TaIPK1, TaIPK2* were identified as being involved in PA biosynthesis in wheat (Aggarwal et al., 2015). A protein, TaABCC13*,* acts as a PA transporter and showed a pleiotropic effect on seed germination and root development (Bhati et al., 2016). Improving mineral bio-availability is possible by reducing PA expression in grain and also can be achieved through over-expression of the phytase enzyme, which degrades phytic acid and release minerals.

Approaches such as mutagenic treatment and transgenic development were adopted to develop low phytic acid mutant lines of maize, rice, barley and soybean. Maize low phytic acid mutants can be grouped into *lpa1, lpa2*, and *lpa3* mutants; *lpa1* affects transporter proteins that packages PA into vacuoles; *lpa2* impairs the production of the inositol phosphate kinase enzyme (IPK1) that phosphorylates inositol-5-phosphate to PA and *lpa3* impairs myo-inositol kinase that phosphorylates myo-inositol to inositol monophosphate (Singh et al., 2020). Plants with *lpa* mutations produced seeds with normal levels of phosphorus but greatly reduced PA bound phosphorus. Low phytic acid mutants of wheat showed a two-fold increase in grain Zn and Fe concentrations (Guttieri et al., 2004; Kenzhebayeva et al., 2019). In mutant lines, the distribution of grain P was altered, increasing the P content in the central endosperm and lowering it in the aleurone layer. But negative pleiotropic effects in *lpa* mutant line Js-12-LPA included reduced yield and height and weak straw, thus limiting the promise of *lpa* mutation for breeding (Guttieri et al., 2004).

Increasing grain phytase activity can readily increase nutrient bioavailablility. Endogenous phytase is produced during seed germination and releases the P from the PA, but in dry seed and flour, digestive tract phytase activity is very low or absent. Overexpression of the *Aspergillus niger* phytase gene (*phyA*) resulted in a 4-fold increase in phytase activity in wheat (Brinch-Pedersen et al., 2000). Detailed studies about increasing phytase activity in wheat are not available. Orthologous genetic information from other related species can be used to identify the gene families involved in PA biosynthesis and degradation and facilitate their use to develop wheat cultivars whose grain features low levels of phytic acid.

## Breeding for improved grain zinc and iron content in wheat

### Genetic analysis of grain zinc and iron in wheat

Understanding the nature of gene action and inheritance for micronutrient accumulation in the wheat grain is a prerequisite for improving the trait. Genetic analysis revealed the quantitative nature of inheritance for grain Zn and Fe contents, making improvement through conventional breeding is slow. Few studies exist regarding the nature of gene action for grain micronutrient content in wheat. One of the studies reported the predominance of additive gene action for grain Fe and dominance and duplicate epistasis for grain Zn in bread wheat (Amiri et al., 2020). Holasoua et al. (2021) reported additive gene action as being significant for both grain Zn and Fe. Higher heritability is paramount for increased genetic gains through selection. High heritability for grain Fe and moderately low heritability for grain Zn were reported by Amiri et al. (2020). Moderate-to-high heritability was detected in other studies (Velu et al., 2016a; Narendra et al., 2021). Low heritability for grain Zn and Fe content was also reported by Joshi et al. (2010). The existence of moderate-to-low heritability and the predominance of dominance and duplicate epistasis suggests advanced generation selection for the traits. High genotype × environment (G × E) interaction for grain Zn and Fe was reported under multilocation testing (Joshi et al., 2010; Velu et al., 2016a). Numerous factors affect grain Zn and Fe content, including soil nutrient availability (Alloway, 2009), soil moisture and organic matter (Cakmak, 2008; Pal et al., 2009), pH (Kirk and Bajita, 1995; Rupa and Tomar, 1999), tillage (Stipesevic et al., 2009), and soil N availability (Singh et al., 2018). Wheat grown under stress conditions showed higher grain Zn and Fe concentrations than crops grown under optimum conditions. Elevated temperatures and drought stress at post-anthesis increased grain Zn concentrations in wheat (Velu et al., 2016a; Narendra et al., 2021). This may be a result of the production of smaller grains and an increased aleurone: endosperm ratio under stress, particularly given that the overall Zn and Fe yield per unit area was higher in non-stressed environments (Velu et al., 2016a). For iron, there was no significant change in grain concentrations between genotypes grown under stressed or optimum conditions (Narendra et al., 2021).

A significant association between grain Zn and Fe contents has been observed (Velu et al., 2016a; Velu et al., 2019; Narendra et al., 2021), indicating improvement in micronutrient levels of one will lead to improvement in the other. This may be due to commonly associated proteins and enzymes for Zn and Fe uptake and translocation to the grains. However, studies have also found a negative association of grain Zn and Fe with grain yield (Morgounov et al., 2007; Narendra et al., 2021). Velu et al. (2016a) reported a negative association of grain yield with grain Zn but no association with grain Fe content. Simultaneous improvement of both traits is challenging. The significant negative association of grain yield with mineral concentrations could be due to a dilution effect, where high-yielding genotypes are contributing less grain Zn and Fe than photosynthates to the grain. In a study comparing modern wheat and rice cultivars with the cultivars released 50 years earlier, it was found that modern cultivars have less capacity to sequester Zn and Fe in the grain than earlier cultivars (Debnath et al., 2021). A study by Hao et al. (2021) found that wheat landraces carry higher grain Zn contents than cultivars and that landraces accumulated more grain Zn under foliar applications of the mineral.

With intensive breeding efforts, high-yielding, mineral dense wheat genotypes can be developed. An example is BARI Gom 33, developed by CIMMYT and released for commercial cultivation in Bangladesh and which offers a 7–8 ppm Zn advantage (Velu et al., 2019), as well as resistance to wheat blast (Roy et al., 2021b). Identification of high-yielding wheat genotypes with higher root uptakes or with a higher capacity to remobilize stored minerals or both is important. Grain Fe was improved with the supply of N in soil and foliar application (Aciksoz et al., 2011b), indicating the presence of useful genes for a higher uptake of N and Fe. In addition, a significant positive association of grain protein content with grain Zn and Fe has been reported (Morgounov et al., 2007). The locus *TaNAM-B1* was detected to correspond higher levels of grain protein, Zn, and Fe (Uauy et al., 2006).

### Use of wild relatives in genetic improvement of grain Zn/Fe

Interestingly, wild species, landraces and synthetic hexaploid wheats (SHW) are probable sources of grain iron and zinc in wheat. Monasterio and Graham (2000) evaluated around 3000 CIMMYT genotypes for grain Zn and Fe concentrations. *T. tauschii* genotypes had average and maximum iron concentrations of 76 and 99 ppm, with an average and maximum zinc concentrations of 50 and 68.9 ppm, respectively. *T. monococcum* had maximum iron and zinc levels of 70 and 131 ppm, respectively. *T. dicoccoides* and *T. dicoccon* showed zinc levels of 142 and 135 ppm. A core collection of Asian bread and durum wheat in the CIMMYT gene bank showed grain Zn levels ranging from 16.85 to 60.77 ppm and, for grain Fe, from 26.26 to 68.78 ppm (Velu et al., 2011). Ninety diploid and tetraploid wheat progenitors were screened for grain Zn and Fe content, few accessions of D and S genome species including *Aegilops tauschii* (D), *Ae. kotschyi* (US), *Ae. speltoides* (S), *Ae. longissima* (S) and *Ae. bicornis* (S) were found promising (Chhuneja et al., 2006). Grain Zn level up to 115.4 ppm (Syn43 [*T. durum* (Yuk) · *Ae. tauschii* (864)], 90.4 ppm (*Ae. tauschii* acc. 14,129) and grain Fe of 109.4 ppm (*Ae. tauschii* acc. 14,102) and 104.4 ppm (*Ae. kotschyi* acc. 3,573) were recorded. Another study reported non-progenitor wild species with S, U and M genome carrying 3–4 times higher grain Zn and Fe than the bread and durum wheat (Rawat et al., 2009b). Tetraploids showed higher concentrations of grain Zn and Fe than hexaploids, with ranges for Zn of 45–177 ppm in *T. boeoticum*, 20–159 in *T. dococcoides*, 29–89 ppm in *T. monococcum* and, for grain Fe, 41–92 ppm in *T. boeoticum*, 28–78 ppm in *T. dococcoides*, and 34–85 ppm in *T. monococcum.* This compared to 15–61 ppm for grain Zn and 24–51 ppm for grain Fe in *T. aestivum* and 18–50 ppm for grain Zn and 10–50 ppm for grain Fe in *T. durum* (Cakmak et al., 2000). Other studies have also reported the high genetic potential for grain Zn and Fe contents in diploid and tetraploid wild species such as *T. monococcum*, *T. turgidum* ssp *dicoccoides*, *T. boeoticum, Aegilops tauschii* and landraces (Cakmak, 2007; Velu et al., 2014). Grain Zn in *T. turgidum* ssp *dicoccoides* varied from 69 to 139 ppm; Fe concentrations from 44 to 88 ppm and protein from 164 to 382 g/kg (Peleg et al., 2007). A few accessions of *T. turgidum* ssp *dicoccoides* showed both high grain Zn and Fe concentrations. In most cases, the cultivated wheat genotypes showed lower levels of grain Zn and Fe. A range of grain Zn and Fe was 21–35 ppm and 22–34 ppm, respectively (Tang et al., 2008), 26–40 ppm and 35–56 ppm, respectively (Peterson et al., 1986), 29–46 ppm and 34–66 ppm, respectively (Ficco et al., 2009), 8–12 ppm and 29–38 ppm, respectively (Cakmak, 2000) were reported in the cultivated wheat genotypes.

Genomic regions associated with higher micronutrient density can be transferred from wild relatives to cultivated varieties. Wheat secondary and tertiary gene pools can be used to broaden the genetic base for grain mineral density through pre-breeding or the production of synthetic hexaploid wheat useful for the breeders. Chromosome addition lines from *Ae. peregrina, Ae. longissima* and *Ae. umbellulata* have been found to carry genes for high Zn and Fe in the grain and roots and control the release of high levels of mugineic acid in the root zone (Neelam et al., 2012). Synthetic amphidiploids produced from *T. aestivum* (AABBDD) and *Ae. kotschyi* (UUS^1^S^1^) carrying grain Zn and Fe more than double of the hexaploid parents (Rawat et al., 2009a).

The addition of rye chromosomes 1R and 7R improved the Zn efficiency of wheat cultivars. Increased zinc concentrations and contents were found in the shoots of chromosome addition lines (Cakmak et al., 1997). One of the most significant achievements in wheat breeding is the addition to hexaploid wheat of the 1BL:1RS rye chromosome segment, which has proved an invaluable source of resistance to stem, leaf, and yellow rust and powdery mildew, as well as providing wide adaptation, enhanced yield, and abiotic stress tolerance (Kumar et al., 2003). Likewise, the 2NS segment from *Aegilops ventricosa* is associated with cyst nematode resistance (Williamson et al., 2013), lodging resistance (Singh et al., 2019), and resistance to wheat blast, one of the devastating diseases of wheat (Roy et al., 2021b; Singh et al., 2021; Phuke et al., 2022). A study using *Ae. longissima* × *T. turgidum* ssp *durum* crosses showed that amphidiploids received genes from *Ae. longissima* for higher grain Zn and Fe contents than their durum parents (Tiwari et al., 2008). Considering the importance of wild relatives as a source of important genes for cultivated wheat, CIMMYT started production of SHW during the 1980s and many of these synthetics provided enhanced resilience against biotic and abiotic stresses (Mujeeb-Kazi, 1995). SHW developed from crossed of *T. turgidum* ssp *turgidum* x *T. tauschii*, *T. turgidum* ssp *durum* x *T. tauschii,* and *T. turgidum* ssp *dicoccoides* x *T. tauschii* produced higher grain Zn and Fe than parental accessions (Zhao et al., 2017). SHW developed by CIMMYT using *T. spelta, T. turgidum* var *diccocoides, and Ae. squarosa* were found to be promising for grain mineral concentration (Calderini and Ortiz-Monasterio, 2003; Crespo-Herrera et al., 2016). Calderini and Ortiz-Monasterio (2003) reported SHW carrying 25%–30% higher grain Zn, Fe and manganese (Mn). Using SHW, several novel genes responsible for high grain Zn and Fe have been transferred into CIMMYT elite wheat breeding lines; some were tested in Bangladesh, India, and Pakistan and released for commercial cultivation (Khokhar et al., 2018; Velu et al., 2018).

### Effect of *GPC-B1* locus on GZn and GFe in wheat

Attempts to detect the genomic regions associated with variation for grain protein, zinc and iron in cultivated and wild relatives of wheat have identified the relevant chromosome locations. For grain protein content, the locus *GPC-B1* was mapped on chromosome 6B (*DIC-6B*) in the durum wheat genotype LND, a D genome disomic substitution line of durum wheat variety LANGDON (LND) from *T. turgidum var dicoccoides* (accession FA-15-3) (Joppa et al., 1997). The *DIC-6B* allele encodes a protein specific to the NAC transcription factor family and was found to be similar to the *Arabidopsis* No Apical Meristem (NAM) protein; therefore, it has been named *NAM-B1* (Uauy et al., 2006). The NAC transcription factor is associated with auxin signaling, responses under biotic and abiotic stress, and early leaf senescence. This pleiotropic gene also increases grain protein, Zn, and Fe and triggers early senescence in the plant. Cultivated wheat carries a non-functional allele of the same gene with a 1 bp insertion of thymine base at position 11 that caused a frameshift mutation (Uauy et al., 2006). The presence of the *DIC-6B* locus increased durum wheat grain Zn, Fe, and Mn levels by 12, 18, and 29%, as well as resulting in a 38% higher protein accumulation than occurs in durum wheat without the locus (Distelfeld et al., 2007). Discovery of high-throughput, tightly-linked markers for *GPC-B1* facilitated the marker-assisted transfer into the cultivated wheat. Through marker assisted breeding, the *GPC-B1* locus was transferred into the Indian wheat variety HUW 468; some derivative lines showed significantly higher levels of grain Zn, Fe, and protein (Vishwakarma et al., 2014). Use of the *DIC* allele was a breakthrough for quality improvement in wheat worldwide. Wheat varieties with more than 14% GPC using the *DIC* locus were developed in Canada (https://www.grainscanada.gc.ca/en/grain-research/scientific-reports/pdf/canadian-wheat.pdf) and Australia (https://seedworld.com/australian-researchers-develop-high-protein-wheat/). Cultivars “Lassik” and “Farnum” in hexaploid wheat and “Westmore” and “Desert King High Protein” in durum wheat in United States; and “Lallian,” “Somerset” and “Burnside” in hexaploid wheat in Canada have been developed (Balyan et al., 2013). Recently, Indian scientists have developed a biofortified wheat variety MACS 4028 that contains 14.7% GPC and above 40 ppm zinc and iron (https://dst.gov.in/scientists-ari-pune-develop-biofortified-high-protein-wheat-variety). Major constraints on the expression of *GPC-B1* appear to be a genetic background and environmental conditions, particularly high temperatures (Carter et al., 2012), making breeding to develop high-yielding, biofortified genotypes using the locus is a challenge, particularly for areas where terminal heat and drought are constraints and may lead to early senescence.

### Mapping of QTLs for high Grain Zn/Fe content in wheat

Selecting genotypes for highly quantitative traits that also show high G × E interaction is difficult through phenotypic assessment. QTL detection and their use through marker assisted selection can improve selection efficiency. The high environmental influence, narrow range of variation, and tedious phenotyping procedures for estimating grain Zn and Fe have made QTL mapping challenging. Phenotyping through inductively coupled plasma spectrometry (ICP-S) and, recently, use of energy-dispersive X-ray fluorescence spectrometry (ED-XRF) has facilitated the screening of large samples, then the atomic absorption spectrometry (AAS).

Efforts have been made in the last two decades to dissect the genetic components governing grain Zn and Fe content in wheat. A number of QTLs associated with the genetic variation of grain mineral content have been detected. QTL mapping was performed on the diverse genetic backgrounds using bi-parental and association mapping population and several QTLs of major and minor allelic variations were detected (Table 2). Most of the QTL mapping studies have focused on the background of SHW or wild relatives of tetraploid or diploid species, due to the presence of higher levels of genetic variation. One early study has reported the *GPC-B1* locus, which has been cloned and sequenced (Uauy et al., 2006). Peleg et al. (2009) used recombinant inbred lines (RILs) developed from *T. durum* (cv. Langdon) × wild emmer wheat (accession #G18-16) and identified 82 QTLs for different minerals, six of which were associated with grain Zn and located on chromosomes 2A, 5A, 6B, 7A, 7B, and 11 of which linked to grain Fe were found on chromosomes 2A, 2B, 3A, 3B, 4B, 5A, 6A, 6B, 7A, and 7B. Using interspecific crosses between *Triticum boeoticum* (pau5088) × *Triticum monococcum* (pau14087), 2 QTLs for grain Fe were detected on chromosomes 2A and 7A and 1 QTL for grain Zn on 7A (Tiwari et al., 2009). Genes for enhanced grain Zn and Fe content were observed on chromosome 2S and 7U of *Ae. kotschyi* and substitution of 2S and 7U for the homoeologous A genome increased up to 117.4% grain Fe and 136% grain Zn over *T. aestivum* cv. WH 711 (Tiwari et al., 2010). A number of SHW were identified as potential donors and their underlying QTLs were detected. Using a SHW × *T. spelta* recombinant inbred line population, 12 QTLs for grain Zn and 7 QTLs for grain Fe were detected (Crespo-Herrera et al., 2017). The QTL *Qgrain Zn.cimmyt-7B_1P2* for grain Zn on chromosome 7B, with a maximum phenotypic variation explained (PVE) of 32.7% and QTL *Qgrain Fe.cimmyt-4A_P2* on chromosome 4A with 21.14% for grain Fe can be used in marker assisted breeding. Pleiotropic QTLs can be targeted through marker assisted selection for simultaneous improvement of multiple traits including yield. QTLs detected on chromosomes 2A, 5A, 6B for grain Zn were co-localized for high GPC and 5 loci on chromosomes 2A, 2B, 5A, 6A, and 7B for high grain Fe and GPC, indicating the potential correlated improvement for grain content of both minerals and proteins (Peleg et al., 2009). Other studies have reported common genomic regions. QTL for grain Zn content on 7B was co-localized with a QTL for grain Fe (Crespo-Herrera et al., 2017). Common genetic regions influencing high GPC, Zn and Fe contents were also found on chromosomes 2A and 5A (Peleg et al., 2009) and on 2A (Krishnappa et al., 2017). Although few, QTLs were detected in the hexaploid wheat background is advantageous, as they can be transferred through breeding without linkage drag, cross compatibility issues, or much time. Using RILs from a cross between a Chinese wheat line (with the lineage Hong Hua Mai/. ../Blouk #1) and the commercial bread wheat cultivar Roelfs F2007, 10 QTLs for grain Zn, 9 for grain Fe, 5 for GPC, and 36 for agronomic traits were detected (Liu et al., 2019b). Pleiotropic QTLs for grain Zn and Fe content on chromosome 3D and for grain Zn, GPC and thousand kernel weight were detected on chromosome 2B. Shi et al. (2008) used a double haploid population derived from two winter wheat cultivars and mapped 4 and 7 genomic locations for grain Zn concentration and grain Zn content, respectively. Four QTLs on chromosomes 4A, 4D, 5A and 7A were identified for grain Zn concentration and content and may facilitate simultaneous improvement of grain Zn content and concentration. Surprisingly, collocated QTLs for grain Zn and phosphorus (P) were found on chromosomes 4A and 4D, suggesting the potential for their correlated breeding improvement, even though the elements share an antagonistic relationship for plant uptake in soils.

**TABLE 2:** Mapping population, number of QTLs detected along with their percent of phenotypic variation explained (PVE%) and chromosome carrying the QTLs for grain zinc and iron in wheat.

Trait	Mapping population and number	Parental details	No. of QTLs	PVE (%)	Chromosomes carrying the QTLs	References
Grain Zn	DH (119)	Hanxuan10 × Lumai 14	7	4.6–14.6	1A, 2D, 3A, 4A, 4D, 5A, and 7A	Shi et al. (2008)
	RILs (93)	*T. boeoticum* accession pau5088 × *T. monococcum* accession pau14087	1	18.8	7A	Tiwari et al. (2009)
	DH (90)	RAC875–2 × Cascades	12	92.0 (combining all QTLs)	3D, 4B, 6B, and 7A	Genc et al. (2009)
	RILs (152)	Durum wheat cv. Langdon × wild emmer wheat (accession #G18-16)	6	1–23.0	2A, 5A, 6B, 7A, and 7B	Peleg et al. (2009)
	RILs (185)	*T. spelta* accession H+ 26 (PI348449) × *T. aestivum* cv. HUW 234	5	4.3–16.5	2A, 2B, 3D, 6A, and 6B	Srinivasa et al. (2014)
	RILs (127 for hexaploid and 105 for tertraploid)	*T. aestivum* cv. Adana99 × *T. sphaerococum* cv. 70,711 and *T. durum* cv. Saricanak 98 × *T. dicoccon* cv. MM5/4	10	9–31.0	1B, 1D, 2B, 3A, 3D, 6A, 6B, 7A, and 7B	Velu et al. (2016b)
	RILs (140)	Seri M82 × SHW CWI76364	6	8.3–19.6	4BS, 6AL, and 6BL	Crespo-Herrera et al. (2016)
	RILs (188)	Bubo × Turtur	4	2.86–16.75	1B, 6A, and 7B	Crespo-Herrera et al. (2017)
	RILs (188)	Louries × Bateleur	12	3.3–32.79	1A, 1B, 3B, 3D, 4A, 5B, 6A, 7B, and 7D	Crespo-Herrera et al. (2017)
	RILs (286)	WH 542 × SHW	5	3.2–14.4	2A, 4A, 5A, 7A, and 7B	Krishnappa et al. (2017)
	GWAS (369)	European elite wheat varieties including 355 genotypes of winter wheat and 14 spring wheat genotypes	161	5.5 to 13.7	1A, 1B, 2A, 2B, 3A, 3B, 3D, 4A, 4D, 5A, 5B, 6A, 6B, 7A, and 7B	Alomari et al. (2018)
	GWAS (123)	123 SHWs	13	1.8–14.1	1A, 2A, 3A, 3B, 4A, 4B, 5A, and 6B	Bhatta et al. (2018)
	GWAS (330)	Harvest Plus Association Mapping panel	39	5–10.5	1A, 2A, 2B, 2D, 5A, 6B, 6D, 7B, and 7D	Velu et al. (2018)
	RILs (200)	Roelfs F2007 × Hong Hua Mai/. ../Blouk #1	10	2.71–14.22	1B, 2B, 3A, 3B, 3D, 4B, 5A, 6B, and 7A	Liu et al. (2019b)
	GWAS (167)	*Ae. tauschii* accessions	4	2.59–3.39	2D, 4D, 6D, and 7D	Arora et al. (2019)
	GWAS (246)	Chinese wheat mini core collection	11	2.7–6.6	1B, 2B, 2D, 3A, 3D, 4A, 4B, 5A, 5D, 6B, and 7D	Liu et al. (2020)
	GWAS (330)	Harvest Plus Association Mapping (AM) panel	13	3.7–5.2	1A, 1D, 2A, 2B, 3A, 4A, 5B, and 7A	Cu et al. (2020)
	RILs (254)	Jingdong 8 × Bainong AK58	7	2.2–25.1	1DS, 2AS, 3BS, 4DS, 6AS, 6DL, and 7BL	Wang et al. (2021)
	RILs (190)	Zinc-Shakti × Kachu	27	1.1–8.1	1A, 2A, 4A, 5A, 6A, 7A, 1B, 2B, 3B, 6B, 1D, 2D, 5D, and 7D	Rathan et al. (2021)
	RILs (95)	AS2407 (*Ae. tauschii*. ssp. *strangulate*) ×AS65 (*Ae. tauschii*. ssp*. tauchii*)	1	13.49	2D	Chen et al. (2022)
	GWAS (280)	Indian wheat germplasm	5	5.7–10.9	2B, 5B, 6A, and 7B	Krishnappa et al. (2022)
Grain Fe	RILs (93)	*T. boeoticum* accession pau5088 × *T. monococcum* accession pau14087	2	11.7–12.6	2A, 7A	Tiwari et al. (2009)
	DH (90)	RAC875–2 × Cascades	10	47.0	3D	Genc et al. (2009)
	RILs (152)	durum wheat cv. Langdon × wild emmer wheat (accession #G18-16)	11	2–18.0	2A, 2B, 3A, 3B, 4B, 5A, 6A, 6B, 7A, and 7B	Peleg et al. (2009)
	RILs (185)	*T. spelta* accession H+ 26 (PI348449) × *T. aestivum* cv. HUW 234	5	1.8–27.1	1A, 2A, and 3B	Srinivasa et al. (2014)
	RILs (127 and 105 for the crosses cv. Adana99 × cv. 70,711 and cv. Saricanak98 × cv. MM5/4, respectively)	*T. aestivum* cv. Adana99 × *T. sphaerococum* cv. 70,711 and *T. durum* cv. Saricanak98 × *T. dicoccon* cv. MM5/4	7	9–18.0	1B, 2A, 2B, 3A, 6B, and 7B	Velu et al. (2016b)
	RILs (140)	Seri M82 × SHW CWI76364	10	7.2–14.5	2BL, 2DS, 4BS, 5AL, 5BL, 6Al, 6BL, 6DS, and 7DS	Crespo-Herrera et al. (2016)
	RILs (188)	*T. spelta* cv. Bubo × Turtur (SHW)	3	5.49–10.35	3A, 4B, 5B	Crespo-Herrera et al. (2017)
	RILs (188)	Louries (SHW) × *T. spelta* cv. Bateleur	7	5.79–21.14	2A, 2B, 3B, 4A, 4D, and 5B	Crespo-Herrera et al. (2017)
	RILs (286)	WH 542 × SHW	4	2.3–6.8	2A, 5A, 7A, and 7B	Krishnappa et al. (2017)
	GWAS (369)	European elite wheat varieties including 355 genotypes of winter wheat and 14 spring wheat genotypes	137	5.6–13.9	1A, 1B, 2A, 2B, 3A, 3B, 4A, 5A, 5B, 5D, 6A, 6D, 7B, and 7D	Alomari et al. (2018)
	GWAS (123)	123 SHWs	3	11.2–13.2	1A, 3A	Bhatta et al. (2018)
	GWAS (369)	European elite wheat varieties	137	5.6–13.9	1A, 2A, 3A, 3B, 5A, 5B, and 6A	Alomari et al. (2019)
	GWAS (167)	*Ae. tauschii* accessions	5	1.47–4.03	1D, 2D, 3D, 4D, and 7D	Arora et al. (2019)
	RILs (200)	Roelfs F2007 × Hong Hua Mai/. ../Blouk #1	9	2.10–14.56	1A, 2A, 3B, 3D, 4B, 5A, and 6B	Liu et al. (2019b)
	RILs (254)	Jingdong 8 × Bainong AK58	4	2.3–30.4	3BL, 4DS, 6AS, and 7BL	Wang et al. (2021)
	RILs (190)	Zinc-Shakti × Kachu	23	1.0 to 10.2	1A, 2A, 4A, 6A, and 7A, 1B, 2B, 4B, 5B, 6B, 1D, 2D, and 7D	Rathan et al. (2021)
	GWAS (280)	Indian wheat germplasm	5	12.7–24.1	1A, 3B, 5A, 6A, and 7B	Krishnappa et al. (2022)

Genome wide association studies (GWAS) use potential natural variations to map significant marker trait associations (MTA), and provide the advantages of high resolution, high allele coverage over the bi-parental mapping population, and elimination of the disadvantages associated with RILs, where variation is confined within the two parental lines. Using GWAS in winter and red wheat cultivars, 161 MTAs were detected on 15 wheat chromosomes. The most significant regions were on chromosomes 3A and 5B and are related to transporter proteins such as those of the *ZIP* family and signal proteins of the *MAPK* family (Alomari et al., 2018). GWAS performed using a diverse panel of SHW develop from of *T. durum*, *T. dicoccon*, *T. spelta*, pre-breeding derivatives from *T. polonicum,* and landraces detected 39 QTLs for grain Zn on chromosomes 1A, 2A, 2B, 2D, 5A, 6B, 6D, 7B, and 7D (Velu et al., 2018). QTLs with large effects were detected on chromosomes 2B and 7B and are associated with transcription factors such as zinc finger motifs and metal ion binding protein (phosphatase), all of which has a role in the additional loading of Zn in wheat grains. Multiple studies have reported QTLs for high Zn and Fe concentrations on chromosomes 2A, 2B, 5A, and 7B (Srinivasa et al., 2014; Alomari et al., 2018; Alomari et al., 2019; Krishnappa et al., 2022). Transfer of these QTLs through marker assisted breeding will certainly improve mineral concentrations in wheat grains. However, detection of QTLs with major effects and stable expression across environments remains a constraint for marker-assisted breeding. Stable QTLs reported in some studies will be initial targets for breeding, but few QTLs were reported to be stable across years or locations. Based on across-location evaluations in India and Mexico, Velu et al. (2018) reported 39 stable MTAs (significant across at least three environments) for grain Zn. Crespo-Herrera et al. (2016) reported a QTL on 4BS that appeared across years governing 19.6% of the PVE for grain Zn and with pleiotropic effects on grain Zn and grain Fe. Alomari et al. (2018) reported a single MTA with minor effects on chromosome 3B and expressed in all 3 years of the study. Cu et al. (2020) reported five MTAs on chromosome 5B expressed over the season and having pleiotropic effects on grain Fe, Mn, Cu, and P contents. Recently, a meta-analysis using QTLs from seven different studies of diverse parental combinations (Shariatipour et al., 2021) detected meta-QTLs (MQTL) on chromosomes 1B, 2B, 4A, 5A, 7A, and 7B with MQTL-1, MQTL-5 and MQTL-7 on chromosomes 1B, 7A, and 7B, respectively, were detected with the highest number of initial QTLs. Functional analysis of candidate genes confined in the MQTL revealed that most of the genes were associated with Zn and Fe homeostasis. The meta-QTL analysis is a powerful tool to reduce the confidence interval for the QTLs by integrating information from independent QTL analyses performed by different authors and helps to identify more reliable QTLs.

**FIGURE 1:**
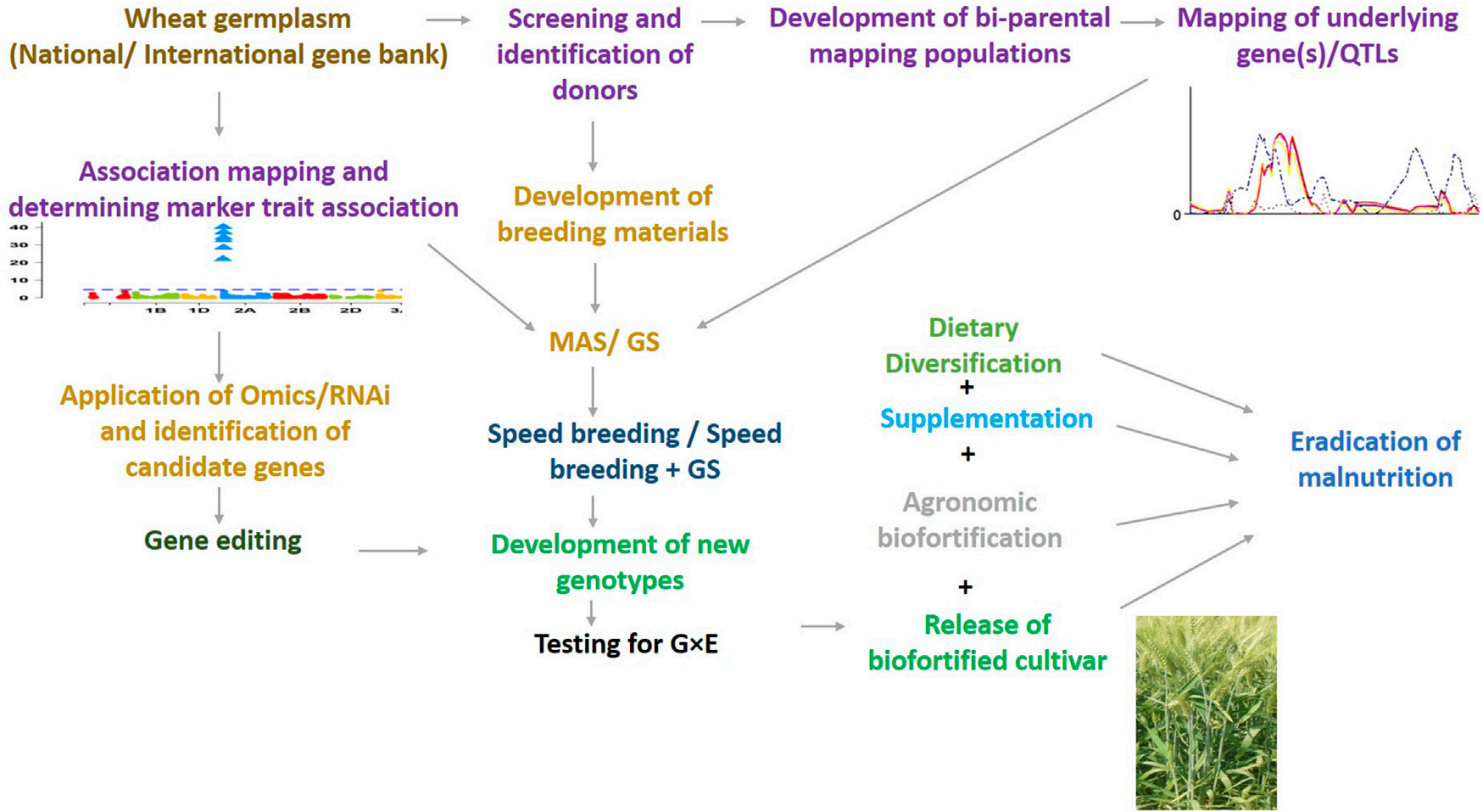
An integrated approach for eradication of malnutrition including new breeding techniques, supplementation and diversification.

Wheat biofortification is challenging, an integrated approach needs to be constructed integrating conventional breeding, biotechnological tools, new breeding techniques, and agronomic measures for immediate soil remediation (Figure 1). In populations with diets heavy in staple crop foods and highly susceptible to micronutrient deficiencies, supplementation through pharmaceutical products and food additives may be required and need support through governmental policies.

## New breeding techniques for biofortification

### Speed breeding

A number of wheat genotypes with higher grain Zn and Fe content have been developed and released for cultivation in Australia, Bangladesh, Bolivia, India, Nepal, Mexico, and Pakistan (Table 3). Constraints associated with selective breeding include limited genetic variation, low heritability, crossability barriers, and linkage drag, to name several. Yield gains through pure line varieties have plateaued and the development of pure line varieties is slow. Working in the joint Rockefeller Foundation-Mexican government Office of Special Studies in the mid-20th century, Nobel Peace laureate Dr. Norman E. Borlaug launched a “shuttle breeding” approach to speed wheat varietal development, allowing two breeding cycles per year and still used by CIMMYT. Double haploids represents another method to rapidly fix genotypes from gametospores of an F_1_ plant, but are also associated with the flaws of a single generation of recombination and not exposed to diverse environments for hardening. Recently, speed breeding has emerged as an approach to the rapid advancement of generation. In this approach, plants are raised in growth chambers under controlled conditions with extended temperatures and photoperiods (Watson et al., 2018). Among other things, this modulates flowering genes to stimulate the early onset of flowering and, as a result, allows 5–6 generations to be grown in a year and the rate of genetic gains is greatly increased. For instance, genetic gain is ∆G = *ihσ*_*A*_ */L*, where *i* = selection intensity, *h* = heritability, *σ*_*A*_ = standard deviation of additive genetic variance and *L* = length of breeding cycle interval or generation. Through modification in the selection intensity and accurate phenotyping, genetic advance can be enhanced to some extent. Rapid breeding cycles also lead to higher genetic gain and the early development of homozygous lines. For quantitative traits with low heritability where the selection in advanced generations is practiced, speed breeding can be of great importance to achieve high homozygosity quickly. Speed breeding has been used in breeding for spring wheat, durum wheat, barley, pea, chickpea and canola (Watson et al., 2018). In wheat, speed breeding has been used to generate genotypes resistant to stem rust, yellow rust, and fusarium head blight (Voss-Fels et al., 2019).

**TABLE 3:** List of biofortified wheat varieties released for commercial cultivation with their grain micronutrient level (ppm), protein content (%) and additional traits (modified from Gupta et al., 2022).

Country origin	Variety	Nutrient level	Year of release	Pedigree	Additional traits
India	WB 02	Zn:42.0, Fe:40.0	2017	T.DICOCCON,CI9309/AE.SQUARROSA (409)//MU-TUS/3/2*MUTUS	Resistance to stem rust (*Sr2*, *Sr7b*), leaf rust (*Lr13*), wheat blast
	DBW 173	Zn:40.7, Protein: 12.5	2018	KAUZ/AA//KAUZ//PBW602	Resistance to stem rust (*Sr31*, *Sr5*), leaf rust (*Lr26*, *Lr10*, *Lr3*), yellow rust (*Yr9*), wheat blast (*2NS*), heat tolerant
	DBW 187	Fe:43.1	2018	NAC/TH.AC//3*PVN/3/MIRLO/BUC/4/2*PASTOR/5/KACHU/6/KACHU	Resistance to stem rust (*Sr5*, *Sr11*), leaf rust (*Lr23*, *Lr10*, *Lr1*), yellow rust (*Yr2*), wheat blast (*2NS*)
	DBW 303	Zn:36.9	2020	WBLL1*2/BRAMBLING/4/BABAX/LR42//BABAX*2/3/SHAMA*2/5/PBW343*2/KUKU NA*2//FRTL/PIFED	Resistance to leaf rust (*Lr13*)
		Fe:35.8			
		Protein: 12.1			
	DDW 47 (d)	Fe:40.1	2020	PBW34/RAJ1555//PDW314	Resistance to stem rust (*Sr11*, *Sr7b*), Yellow rust (*Yr2*)
		Protein: 12.7			
	DDW 48(d)	Zn:39.7	2020	HI8498/PDW233//PDW291	Resistance to stem rust (*Sr7b*, *Sr2*)
		Fe:38.8			
		Protein: 12.1			
	HPBW 01	Zn:40.6	2017	T.DICOCCON CI 9309/A. SQUARROSA (409)/3/MILAN/S87230//BAV92/4/2^*^ MILAN/S87230//BAV92	Resistance to stem rust (*Sr2*, *Sr31*), leaf rust (*Lr10*, *Lr23*, *Lr26*), yellow rust (*Yr9*)
		Fe:40			
	PBW 757	Zn:42.3	2018	PBW550/YR15/6*AVOCET/3/2*PBW550/4/PBW568 + YR36/3*PBW550	Resistance to stem rust (*Sr8a*, *Sr5*, *Sr2*), leaf rust (*Lr13*, *Lr10*, *Lr1*)
	PBW 752	Zn:38.7	2018	PBW621/4/PBW343//YR10/6*AVOCET/3/3*PBW343/5/PBW621	Resistance to stem rust (*Sr13*, *Sr11*), leaf rust (*Lr13*), wheat blast (*2NS*)
		Fe:37.1			
		Protein: 12.4			
	PBW 771	Zn:41.4	2020	PBW550//YR15/6*AVOCET/3/2*PBW550	Resistance to stem rust (*Sr31*), leaf rust (*Lr26*, *Lr23*, *Lr1*) yellow rust (*Yr9*)
	HI 8777 (d)	Zn:43.6	2017	B93/HD4672/HI8627	Resistance to stem rust (*Sr7b*), yellow rust (*Yr2*)
		Fe:48.7			
	HI 1605	Zn:35	2017	BOW/VEE/5/ND/VG9144//KAL//BB/3/YACO/4/CHIL/6/CASKOR/3/CROC_1/AE.SQ (224)//OPATA/7/PASTOR//MILAN/KAUZ/3/BAV92	Resistance to stem rust (*Sr5*, *Sr11*) leaf rust (*Lr13*), yellow rust (*Yr2*)
		Fe:43			
		Protein: 13			
	HI 8759 (d)	Zn:42.8	2017	HI8663/HI8498	Resistance to stem rust (*Sr2*, *Sr11*), leaf rust (*Lr23*)
		Fe:42.1			
		Protein: 12			
	HI 1633	Zn:41.1	2020	GW322/PBW498	High gluten strength (5 + 10 subunit of Glu-D1)
		Fe:41.6			
		Protein 12.4			
	HI 8805 (d)	Fe:40.4	2020	IWP5070/HI8638//HI8663	Resistance to stem rust (*Sr13*, *Sr11*), leaf rust (*Lr13*)
		Protein: 12.8			
	HD 3171	Zn:47.1	2017	PBW343/HD2879	Resistance to stem rust (*Sr11*, *Sr7b*, *Sr2*), leaf rust (*Lr23*, *Lr13*, *Lr10*), yellow rust (*Yr2*), wheat blast, drought tolerant
	HD 3249	Fe:42.5	2020	PBW343*2/KUKUNA//SRTU/3/PBW343*2/KHVAKI	Resistance to stem rust (*Sr11*, *Sr2*), leaf rust (*Lr13*, *Lr10*), yellow rust (*Yr2*), wheat blast (*2NS*)
	HD 3298	Fe:43.1	2020	CL1449/PBW343//CL882/HD2009	Leaf rust (*Lr23*), Yellow rust (*Yr2*)
		Protein: 12.1			
	MACS 4028 (d)	Zn:40.3	2018	MACS2846/BHALEGAON3^*^2	Resistance to stem rust (*Sr7b*)
		Fe:46.1			
		Protein: 14.7			
	MACS 4058	Zn:37.8	2020	MACS3125/AKDW2997-16//MACS3125	Resistance to stem rust (*Sr13*) Leaf rust (*Lr23*)
		Fe:39.5			
		Protein: 14.7			
	UAS 375	Protein: 13.8	2018	UAS 320/GW322//LOK62	Resistance to stem rust (*Sr7b*, Sr2), leaf rust (*Lr13*), yellow rust (*Yr 2*)
Pakistan	NR- 421 (Zincol-2016)	High Zn (>6 ppm Zn advantage compared to best local check)	2015	OASIS/SKAUZ//4*BCN/3/2*PASTOR/4/T.SPELTA PI348449/5/BACEU#1/6/WBLL1*2/CHAPIO	High yield
	Akbar-2019	High Zn (>7 ppm Zn advantage compared to best local check)	2019	Becard/Quaiu	Resistant to yellow rust and wheat blast
	Nawab 2021	High Zn		HGO94.7.1.12/2*QUAIU #1/3/VILLA JUAREZ F2009/SOLALA//WBLL1*2/BRAMBLING	Resistant to yellow rust and wheat blast
Bangladesh	BARI Gom 33	Zn: 50–55	2017	Kachu/Solala	Wheat Blast (*2NS*)
Mexico	Nohely-F2018	7%–8% Zn advantage over check	2018	T.DICOCCON (CI 9309)/AE.SQUARROSA (409)//MUTUS/3/2*MUTUS	Resistance to leaf rust, yield 7.8 t/ha
Bolivia	Iniaf-Okinawa	High Zn (>8 ppm Zn advantage than the local check)	2018	Kachu/Solala	Wheat blast (*2NS*)
Nepal	Zinc Gahun 1 (NL 1327)	High Zn (>6 ppm Zn advantage than the local check)	2020	MELON//FILIN/MILAN/3/FILIN/5/CROC_1/AE.SQUARROSA (444)/3/T.DICOCCON PI94625/AE.SQUARROSA (372)//3*PASTOR/4/T.DICOCCON PI94625/AE.SQUARROSA (372)//3*PASTOR/6/ATTILA/3*BCN//BAV92/3/TILHI/5/BAV92/3/PRL/SARA//TSI/VEE#5/4/CROC_1/AE.SQUARROSA (224)//2*OPAT	High yield
	Zinc Gahun 2 (NL1369)	High Zn	2020	T. DICCOCON CI 9309/AE. SQUARROSA (409)//MUTUS/3/2*MUTUS	High yield
	Bheri-Ganga (WK 2748)	High Zn	2020	MELON//FILIN/MILAN/3/FILIN/5/CROC_1/AE.SQ UARROSA (444)/3/T.DICOCCON PI94625/AE.SQUARROSA (372)//3*PASTOR/4/T.DICOCCON PI94625/AE.SQUARROSA (372)//3*PASTOR	High yield
	Himganga (WK 3026)	High Zn	2020	CHONTE*2/SOLALA//2*BAJ #1	High yield
	Khumal-Shakti (WK 3027)	High Zn	2020	FRNCLN*2/7/CMH83.1020/HUITES/6/CMH79A.9 55/4/AGA/3/4*SN64/CNO67//INIA66/5/NAC/8/WB LL1*2/KURUKU//HEILO/9/WBLL1*2/KURUKU//H EILO	High yield
	Borlaug 2020 (NL 1307)	High Zn	2020	ROLF07/4/BOW/NKT//CBRD/3/CBRD/5/FRET2/TUKURU//FRET2	High yield

d, indicates durum wheat variety

### Genomic selection and integrating genomic selection with speed breeding

Genomic selection (GS) is a powerful tool to increase genetic gains, shorten breeding cycles, prediction the performance of an individual in an untested environment, and increase selection accuracy and genetic gain. GS is most effective for the traits where phenotyping is costly—particularly, yield and quality traits. GS can be used for the selection of quantitative traits involving complex physiological mechanisms, each underpinned by multiple genetic variations that require genome-wide selection approaches, rather than targeting single QTLs. GS calculates genomic assisted breeding values (GEBVs) by estimating all available genetic variations in an individual. It uses the genomic best linear unbiased prediction (GBLUP) model and a multi-environment, linear mixed model to estimate correlated environmental structure, to predict the performance of individuals before phenotyping, based on genotyping and pedigree information. In GS, a set of individuals are genotyped and phenotyped as a training population for use to develop the prediction model (Juliana et al., 2018). The efficacy of GS has been tested for multiple traits in several crops, including wheat, for yield (Juliana et al., 2018), leaf rust and yellow rust (Juliana et al., 2017), spot blotch (Juliana et al., 2022b), and wheat blast (Juliana et al., 2022a). GS to improve grain mineral concentrations has been employed in maize (Mageto et al., 2020), rice (Rakotondramanana et al., 2022), and wheat (Velu et al., 2018), with reasonable prediction accuracies. Velu et al. (2018) reported prediction accuracies ranging from 0.331 to 0.694, with an average of 0.542, for grain Zn content and 0.324 to 0.734, with an average of 0.529, for grain Fe content, in the Harvest Plus Association Mapping (HPAM) panel. Prediction accuracy was higher in the environments with high heritability and soil available Zn. Genetic relatedness increases prediction ability and prediction ability sharply declines as relatedness between the training and testing population decreases (Crossa et al., 2010) and when evaluations are conducted in poor environments and with poor phenotyping (Velu et al., 2018). GS becomes more effective with higher prediction accuracies and applied in early generations of selection to retain lines with high breeding value and discarding those not expected to give higher genetic gains in advanced generations. GS can easily be incorporated with speed breeding to increase genetic gain. GS has not been studied in detail for increasing mineral density in wheat. A model has been proposed where GS was accumulated through speed breeding and predicted higher genetic gains over conventional breeding (Voss-Fels et al., 2019). It has been shown that the incorporation of GS with speed breeding can significantly increase grain yield, over conventional phenotypic selection.

### Genome editing

Genome editing is a powerful tool in the field of medicine, agriculture and the life sciences, allowing targeted changes in a genotype and avoided the random changes that occur in induced mutations. Genome editing can produce targeted genetic variation where none previously existed. Unlike the case of genetically modified crops, gene editing does not require the insertion of foreign DNA. Deletions, additions, single nucleotides or DNA segment substitution are used to change a target gene. Researchers have developed artificial site-specific nucleases, broadly classified as zinc-finger nucleases (ZFNs), transcription activator-like effector nucleases (TALENs), and clustered regularly interspaced short palindromic repeats (CRISPR)-CRISPR-associated (Cas) nucleases that are designed to effect a targeted, double-stranded break in the DNA (Karmakar et al., 2022). ZFNs consist of a zinc finger DNA binding domain attached with nuclease FokI (Carroll, 2011). TALENs are engineered with a TAL effector binding DNA domain attached with FokI nuclease (Joung and Sander, 2013). CRISPR-Cas nuclease consists of a guide RNA and a Cas nuclease. Using the Cas9 endonuclease, CRISPR-Cas9 is the most extensively used system for genome editing, thanks to its simple design, cost effectiveness, high efficacy, reproducibility and engineering feasibility (Karmakar et al., 2022). A single guide RNA (sgRNA) hybrid consisting of a CRISPR-RNA and a transactivating RNA locate the binding and cleavage site for the Cas9 nuclease. CRISPR-Cas9 genome editing is increasingly used to enhance disease resistance, tolerance to abiotic stresses (drought, heat, salinity) and end-use quality in food crops. The role of genome editing in crop biofortification is being exploited for several traits including vitamin-A enrichment, targeted increases in grain zinc and iron, and reducing anti-nutritional factors in the grain (Kumar et al., 2022). Targeted knockout of the *OsAAP6* and *OsAAP10* genes using gene editing has reduced protein content in rice (Wang et al., 2020). The CRISPR-Cas9 system was used to create novel *OsBADH2* genes in non-aromatic rice lines and created aroma (Kumar et al., 2020). Targeted change in *OsNRAMP2* gene enhanced iron remobilization and distribution in rice (Chang et al., 2022). In wheat, CRISPR-Cas9 mediated genome change in the *α-gliadin* gene resulted in low gluten wheat (Sánchez-León et al., 2018). Reduction in phytic acid levels through the disruption of inositol penta*kis*phosphate 2-kinase 1 (*TaIPK1*) improves grain Zn and Fe bioavailability in wheat (Ibrahim et al., 2021). In the future, numerous genes can be targeted through CRISPR-Cas to develop biofortified crops. To promote genome edited technology in crop improvement, Argentina, Brazil, Colombia, Chile, India, and the United States have established regulatory measures separate from those applied for transgenic crops.

### RNAi technology

The suppression of gene expression through antisense or RNAi technology is a powerful way to modulate biosynthetic pathways. RNAi is preferred over antisense technology, as it is more stable, efficient, and precise. Using a double stranded RNA (dsRNA) molecule, RNAi inhibits the expression of a gene at the transcription and translational levels. It has been used for biotic and abiotic stress tolerance and nutritional quality improvement. RNAi mediated suppression of the inositol penta*kis*phosphate kinase (*IPK1*) gene involved in the phytic acid biosynthesis pathway reduced phytic acid level of 28%–56% in wheat (Aggarwal et al., 2018). Similarly, downregulation of the rice inositol triphosphate kinase (*OsITP5/6K-1*) gene through RNAi resulted in a 42% reduction in phytic acid in rice grains (Karmakar et al., 2020). This technology can be used to elucidate biosynthetic pathways and understand the roles of different genes in Zn and Fe sequestration.

### Omics technology

Advancement in next-generation sequencing platforms has enabled the development of a whole genome sequence for *Triticum aestivum* cv. Chinese Spring (https://www.wheatgenome.org/News/Latest-news/RefSeq-v1.0-URGI), which is the largest cereal genome. Genome sequence information of diploid (AA, DD genome) and tetraploid (AABB) progenitors are also available (Zhou et al., 2020). Sequence information for wheat wild relatives will elucidate the genetic mechanisms and variation for micronutrient levels in wheat grain, paving the way for structural genomics, functional genomics and metabolomics. Integration of multiple omics data with high-throughput techniques has elucidated the complex pathways of plant growth and their responses under biotic and abiotic stress. The availability of other omics technologies (mutagenomics, pangenomics, transcriptomics, proteomics, ionomics, and phenomics) would provide new knowledge on complex biological systems. Huge amounts of data are generated using these technologies; advances in computing and integrated system-based analysis to use and manage data will expand our understanding of genotype and phenotype relationships, allowing wheat breeders to make targeted changes in the pathways of metal homeostasis and develop bio-fortified genotypes.

## Conclusion

The review updates about wheat biofortification research include conventional and molecular breeding. Mainstreaming biofortification is essential along with yield, disease resistance and other parameters. Being quantitative, limited genetic variations in the cultivated wheat varieties, improvement through conventional breeding is hard. Available genomic resources are needed to be utilized while breeding for high grain zinc and iron content wheat varieties. An integrated breeding programme including marker assisted selection, genomic approaches and gene editing tools will gear up development of biofortified wheat varieties.
